# Saline-alkali gradients reshape soil microbial network complexity and niche breadth

**DOI:** 10.3389/fmicb.2026.1886660

**Published:** 2026-06-29

**Authors:** Hanghang Hou, Xiaoling Zhang, Siyuan Chen, Yuyao Kong, Shangkun Yang, Zhijun Gao, Zhijia Cui, Ziyang Lv, Zhengwei Yang, Yuhao Yuan, Baili Feng

**Affiliations:** 1 State Key Laboratory of Crop Stress Biology in Arid Areas, College of Agronomy, Northwest A&F University, Yangling, China; 2 Inner Mongolia Erdos Institute of Agriculture and Animal Husbandry, Erdos, China; 3 College of Agronomy, Henan Agricultural University, Zhengzhou, China

**Keywords:** generalist, microbial network, niche breadth, saline-alkali, specialist

## Abstract

**Introduction:**

Saline-alkali soils impose combined osmotic, ionic and alkaline constraints on soil microorganisms, yet how bacterial and fungal ecological strategies vary along saline-alkali gradients remains insufficiently resolved.

**Methods:**

We analyzed 30 composite soil samples from 10 sites across China using bacterial 16S rRNA and fungal ITS amplicon sequencing, soil physicochemical profiling, co-occurrence network analysis, niche breadth classification and *PICRUSt2*-based functional prediction.

**Results:**

Higher saline-alkali intensity was associated with reduced nutrient availability, lower microbial network complexity and greater network vulnerability. Bacterial specialists showed stronger diversity and compositional responses than generalists, whereas fungal communities displayed comparatively stable patterns across the sampled gradient. Predicted bacterial functional profiles suggested an increased representation of stress-survival-related pathways under high saline-alkali conditions.

**Discussion:**

These findings identify microbial taxa, network properties and predicted functional features associated with saline-alkali soil degradation and provide candidate targets for future culture-based, metagenomic and experimental validation.

## 1 Introduction

Saline-alkali soils are a major form of salt-affected land degradation, affecting approximately 1.38 billion hectares worldwide and threatening agricultural productivity and food security (FAO, 2024). They are characterized by the accumulation of soluble salts, elevated alkalinity and sodium-related ionic imbalance, which jointly degrade soil structure, constrain nutrient availability and alter biological functioning (Zaman et al., 2018; Richards et al., 1954). These changes can reduce crop yields and disrupt microbial diversity and ecological processes in soil ecosystems (Kumar et al., 2024). Over the past decades, various remediation strategies have been developed worldwide, including hydrological regulation, physical amendment, engineering-based salt drainage, and the application of chemical soil conditioners. While these approaches have achieved localized success, they are often limited by factors such as high costs, poor long-term sustainability, and environmental risks (Wang et al., 2024). In recent years, biological remediation has attracted considerable attention as an environmentally friendly and sustainable alternative for improving salt-affected soils (Kumar Arora et al., 2020).

Soil microorganisms regulate organic matter turnover, nutrient cycling and plant stress responses, and their activities are closely linked to soil physicochemical properties and plant performance (Philippot et al., 2024; Trivedi et al., 2020). In saline-alkali soils, microbial communities are exposed to combined osmotic pressure, ionic imbalance and alkaline constraints, which can alter microbial growth, resource acquisition and community assembly (Sliti et al., 2025; Zaman et al., 2018). More broadly, microbiome responses to environmental change can involve ecological and evolutionary processes that alter community structure and functional potential (Martiny et al., 2023). Therefore, microbial responses to saline-alkali conditions are unlikely to be fully captured by taxonomic composition alone. Co-occurrence networks, niche breadth and predicted functional profiles can provide complementary information on how microbial communities reorganize under environmental stress, including changes in association patterns, ecological specialization and potential metabolic strategies (Kajihara and Hynson, 2024; Yuan et al., 2021). Understanding these ecological responses is important for identifying microbial indicators of saline-alkali soil degradation and for selecting candidate taxa or functions for future soil restoration studies.

Microbial taxa differ substantially in niche breadth and environmental tolerance. Habitat generalists can persist across a wide range of environmental conditions, whereas specialists are restricted to narrower ecological niches and are often more sensitive to environmental filtering (Xu et al., 2022; Levins, 1968). This generalist-specialist framework has been increasingly used to interpret microbial biogeography, community assembly and functional differentiation under environmental stress (Du et al., 2025). In saline-alkali soils, such niche differentiation may be particularly important because salt accumulation, alkalinity and sodium-related ionic imbalance can create strong environmental filters. However, it remains unclear whether bacterial and fungal generalists and specialists respond similarly along saline-alkali gradients, and whether changes in niche breadth are accompanied by changes in network complexity and predicted functional potential.

Functional prediction provides an additional perspective for evaluating whether taxonomic and ecological shifts are associated with changes in microbial functional potential. In degraded or nutrient-limited soils, microbial communities may retain some predicted core functional capacities despite changes in community composition, a pattern often discussed in relation to functional redundancy or potential compensation (Liu S. et al., 2025; Li Y. et al., 2024; Trivedi et al., 2020). Marker-gene-based prediction tools such as *PICRUSt2* can infer potential functional profiles from 16S rRNA data, although such predictions should be interpreted cautiously because they do not directly measure genes, transcripts or metabolic activity (Douglas et al., 2020; Yang et al., 2023). Whether predicted functional shifts occur consistently across saline-alkali gradients, and whether they are linked to bacterial and fungal generalist-specialist differentiation, remains unresolved.

To address these gaps, we examined soil physicochemical properties, bacterial and fungal community composition, co-occurrence networks, niche breadth patterns and predicted bacterial functional potential across low, moderate and high saline-alkali soils in China. We asked whether increasing saline-alkali intensity was associated with reduced nutrient availability, lower network complexity, divergent responses of generalists and specialists, and shifts in predicted functional profiles. Because this study was based on field sampling across geographically separated sites, we interpret these patterns as associations with measured saline-alkali soil conditions rather than as direct evidence of a single causal driver. This design allowed us to identify microbial ecological patterns linked to saline-alkali gradients and to define candidate taxa, network properties and predicted functions for future metagenomic and experimental validation.

## 2 Materials and methods

### 2.1 Experiments and soil sampling

**Figure 1:**
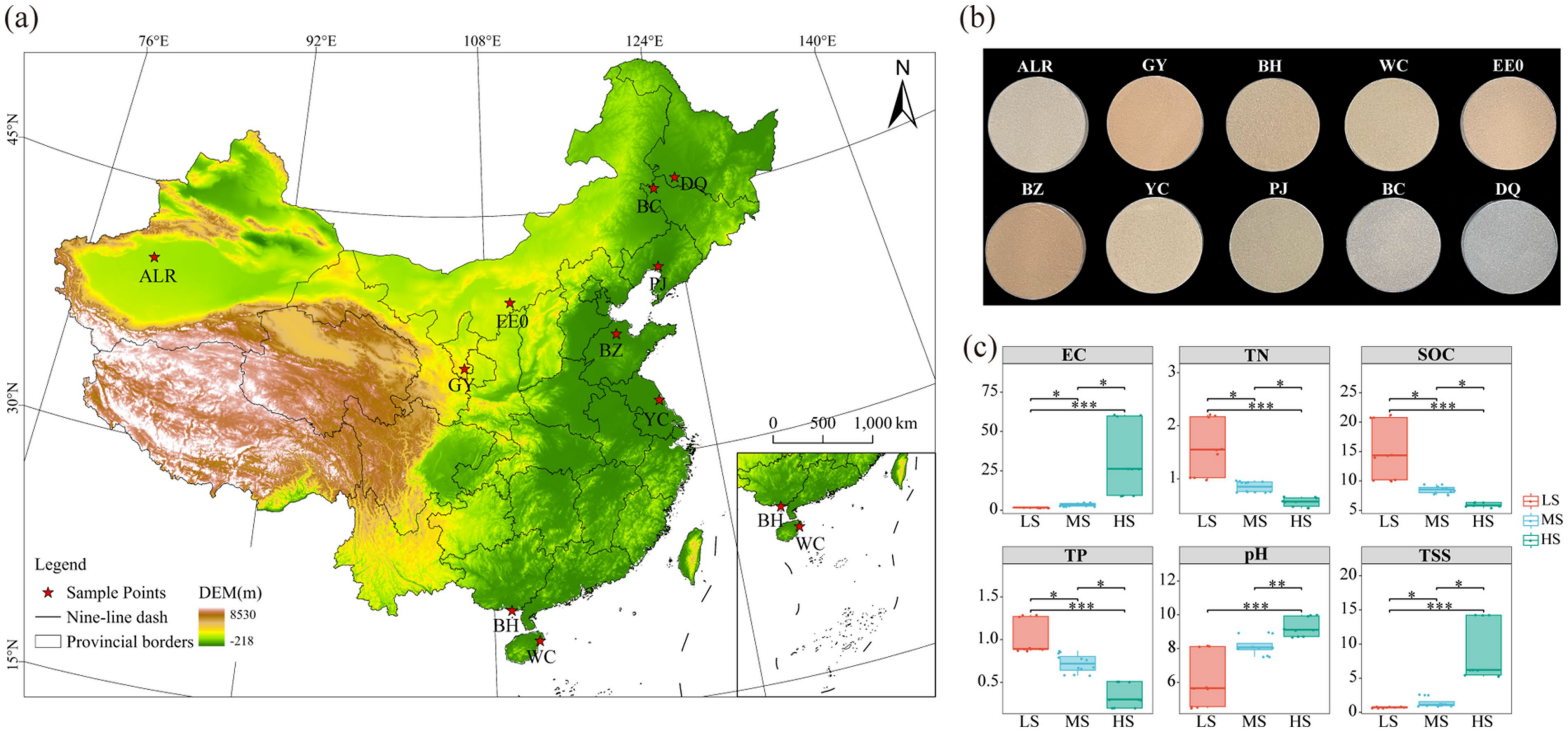
Geographical locations and soil physicochemical properties of soil samples collected in this study. **(a)** Locations of sampling sites; **(b)** soil samples from each sampling site; **(c)** soil physicochemical properties across different groups. EC, electrical conductivity; TN, total nitrogen; SOC, organic carbon; TP, total phosphorus; pH, soil pH; TSS, total soluble salts. *p < 0.05, **p < 0.01, ***p < 0.001.

We investigated a 3,547 km east–west soil longitudinal gradient (20°02′ - 46°35′N, 81°22′ - 125°10′E), spanning 10 provinces across China (Figure 1a). Soil samples were collected from 10 sites between July and August 2022. At each site, three randomly selected 30 m^2^ plots were designated for sampling. Within each plot, soil samples were collected from five sampling points at a depth of 0–20 cm and combined to obtain a composite sample for each plot, resulting in a total of 30 composite soil samples. A portion of each composite sample was transferred into a 50 mL sterilized centrifuge tube and immediately transported to the laboratory in a portable, truck-mounted refrigerator (−20 °C) to maintain sample integrity. The composite soil samples were subsequently sieved through a 0.1 cm mesh and divided into two subsamples. One subsample was stored at 4 °C for soil property analysis, and the other one was preserved at −80 °C for subsequent DNA extraction. Following previous salinity assessment frameworks (Zaman et al., 2018; Richards et al., 1954), we used soil EC as the primary grouping variable because EC directly reflects soluble salt accumulation and showed the clearest separation among the sampled sites. The sampling sites were therefore assigned to three operational EC-defined saline-alkali intensity groups, rather than formal saline, sodic or saline-sodic soil classes. The EC ranges were 0.15–0.18 dS·m^−1^ for LS, 0.24–0.50 dS·m^−1^ for MS and 0.93–6.03 dS·m^−1^ for HS. The high-saline-alkali (HS) group comprised samples BC (Baicheng City, Jilin Province), PJ (Panjin City, Liaoning Province), and YC (Yancheng City, Jiangsu Province), the moderate-saline-alkali (MS) group included samples GY (Guyuan City, Ningxia Province), EE0 (Erdos City, Inner Mongolia), BH (Beihai City, Guangxi Province), and DQ (Daqing City, Heilongjiang Province), and the low-saline-alkali (LS) group encompassed samples WC (Wenchang City, Hainan Province), ALR (Alaer City, Xinjiang), and BZ (Binzhou City, Shandong Province). Other saline-alkali indicators, including pH, SAR, ESP, TSS and K^+^/Na^+^, were not used as grouping variables but were used to characterize alkalinity, sodicity, soluble salt accumulation and ionic imbalance within and among groups. Further details regarding the sample site characteristics are provided in Supplementary Table S1.

### 2.2 Analysis of soil physicochemical properties

The soil organic carbon content (SOC) was determined using the potassium dichromate oxidation method combined with external heating and sulfuric acid digestion (Yang et al., 2022). Total nitrogen (TN) was quantified with a SAN++ continuous flow analyzer following sulfuric acid digestion and colorimetric detection based on the Kjeldahl method (Deng et al., 2020). Total phosphorus (TP) was determined after alkali fusion with sodium hydroxide, followed by dissolution in dilute acid and quantification via molybdenum-antimony colorimetry using the SAN++ analyzer (Cao et al., 2016). Soil pH and EC were assessed in a 1:5 (w/v) soil-to-water suspension after 30 min of equilibration, using a REX PHS-3E pH meter and a DDSJ-319 L conductivity meter (INESA Scientific Instrument Co., Ltd.), following standardized protocols (Yan et al., 2023). The Ca^2+^ and Mg^2+^ concentrations were determined via atomic absorption spectrometry (Pessoa et al., 2022), and the Na^+^ and K^+^ levels were measured using flame emission photometry (Pessoa et al., 2022). The Cl^−^ content was determined by titration with AgNO₃ solution, the levels of HCO₃^−^ and CO₃^2−^ by titration with H₂SO₄, and the SO₄^2−^ level by gravimetric analysis using BaCl₂ (Pessoa et al., 2022). Total soluble salts (TSS) were determined by evaporating a soil-water extract (Lin and Bañuelos, 2015). The cation exchange capacity (CEC) was calculated as the sum of all exchangeable cations (K^+^, Ca^2+^, Na^+^, and Mg^2+^) and expressed in mmolc kg^−1^ (Rosinger et al., 2025). The exchangeable sodium percentage (ESP) was determined as the proportion of exchangeable Na^+^ relative to the cation exchange capacity (CEC), following the formula described by Pessoa et al. (2022):


$$\mathrm{ESP}\;(\%)={\mathrm{Na}}^{+}/\mathrm{CEC}\times 100.$$

The sodium adsorption ratio (SAR) was estimated from soluble ion concentrations using the following equation (Pessoa et al., 2022):


$$\mathrm{SAR}\;{\left({\text{mmol}}_{c}L^{-1}\right)}^{0.5}={\mathrm{Na}}^{+}/[({\mathrm{Ca}}^{2+}+{\mathrm{Mg}}^{2+})/2].$$

### 2.3 Sequencing and bioinformatics analysis

Genomic DNA was extracted from the soil samples using the E.Z.N.A.^®^ Soil DNA Kit (Omega Bio-Tek, Inc., USA) according to the manufacturer’s protocol (Hernandez et al., 2021). The integrity and purity of the extracted DNA were assessed by 1% agarose gel electrophoresis, and the concentration was measured using a NanoDrop^®^ spectrophotometer (Thermo Scientific, CA, USA). All DNA samples were stored at −20 °C until further analysis. For bacterial community profiling, the V3-V4 hypervariable region of the 16S rRNA gene was amplified using the universal primers 338F (5’-ACTCCTACGGGAGGCAGCAG-3′) and 806R (5’-GGACTACHVGGGTWTCTAAT-3′), with unique 8-bp barcodes attached to the 5′ ends of both primers for samples identification (Fadrosh et al., 2014). The PCR amplification was conducted on an ABI 9700 thermocycler (Applied Biosystems, USA) under the following conditions: initial denaturation at 37 °C for 15 min, 5 cycles of 98 °C for 30 s, 98 °C for 10 s, and 65 °C for 75 s, and a final extension at 65 °C for 5 min. Finally, the samples were stored at 4 °C. Fungal community composition was assessed by amplifying the internal transcribed spacer (ITS) region using primers ITS1F (5′-CTTGGTCATTTAGAGGAAGTAA-3′) and ITS2R (5’-TGCGTTCTTCATCGATGC-3′), following the protocol provided by Luan et al. (2015). The PCR conditions for fungal ITS amplification was identical to those used for the bacterial 16S rRNA gene. All purified amplicons were sequenced using the Illumina MiSeq PE300 platform (Illumina, Inc., USA) at Beijing Allwegene Gene Technology Co., Ltd., following the manufacturer’s recommendations (Yuan et al., 2023). Raw paired-end reads were demultiplexed according to sample-specific barcodes. Primer and barcode sequences were removed before downstream processing. Raw reads were filtered and merged using PEAR v0.9.6. Reads containing ambiguous bases were discarded, and low-quality regions with quality scores ≤ 20 were trimmed. Paired-end reads were merged with a minimum overlap of 10 bp and a *p*-value threshold of 0.0001. The merged sequences were further processed using VSEARCH v2.7.1. For bacterial 16S rRNA sequences, reads shorter than 230 bp were removed, and chimeric sequences were identified and removed using the UCHIME method against the Gold database. For fungal ITS sequences, reads shorter than 230 bp were removed, and chimeric sequences were removed using the UCHIME method against the UNITE database. High-quality sequences were clustered into operational taxonomic units (OTUs) at 97% sequence similarity using the UPARSE algorithm implemented in VSEARCH v2.7.1. Representative OTU sequences were taxonomically assigned using BLAST. Bacterial 16S rRNA representative sequences were classified against the SILVA 138 database, whereas fungal ITS representative sequences were classified against the UNITE 8.2 database. The e-value threshold for taxonomic assignment was set to 0.00001. To reduce the influence of unequal sequencing depth among samples, all samples were rarefied to the same sequencing depth before downstream analyses. After quality filtering and OTU selection, 16,910 bacterial OTUs and 4,454 fungal OTUs were retained for subsequent analyses.

### 2.4 Network construction and network complexity and stability analyses

To investigate the impacts of saline-alkali and soil heterogeneity on the soil microbial community structure, we constructed three meta-communities for each sampling locality, incorporating both bacterial and fungal OTUs. Spearman correlation coefficients among OTUs within each meta-community were computed using the *Hmisc* package in R (Langfelder and Horvath, 2008). Based on these correlation matrices, co-occurrence networks were generated, retaining only associations with absolute correlation coefficients (|r|) > 0.8 and a false discovery rate (FDR)-adjusted *p* < 0.01. Networks were generated using the *graph_from_adjacency_matrix* function from the *igraph* package, where nodes represented individual OTUs and edges denoted statistically significant associations. The OTUs without significant interactions were excluded from downstream analyses. Positive and negative edges were interpreted as statistical co-occurrence and co-exclusion patterns, respectively, rather than as direct evidence of mutualistic or competitive interactions. A total of 18 empirical microbial co-occurrence networks were visualized using Gephi (v. 0.9.2; Bastian et al., 2009; Wang et al., 2023). To assess the significance of the observed network structures, we generated 100 randomized networks per locality by arbitrarily rewiring the edges among nodes using functions in the *igraph* package. To characterize the topological structures of the microbial networks, a comprehensive set of network metrics was calculated using *igraph* (the metric definitions are detailed in Supplementary Table S2, and the results are summarized in Supplementary Table S3) (Wang et al., 2023). In addition, we computed the relative modularity of each network following the approach described by Thébault and Fontaine (2010), accounting for differences in network size and complexity among communities. Here, network complexity refers to the diversity and richness of microbial interactions, ranging from mutualistic to antagonistic and is regarded as a meaningful indicator of ecological functioning (Wang et al., 2023). Community stability was assessed using multiple complementary indices. First, we calculated the natural connectivity to quantify network robustness under simulated disturbance scenarios. This metric, which reflects a network’s resilience to species loss, was calculated by iteratively removing nodes at random (Peng and Wu, 2016). Second, network vulnerability was evaluated by identifying the maximum vulnerability value among nodes, which indicates the extent to which each node influences overall network efficiency (Yuan et al., 2021). Third, we examined microbial network cohesion, including total cohesion as well as its positive and negative components. Cohesion refers to the strength of associations among taxa, shaped by ecological interactions or niche similarities and differences (Herren and McMahon, 2017). Specifically, positive and negative cohesion were calculated as the abundance-weighted sums of significant correlations of respective signs, whereas total cohesion was defined as the sum of positive cohesion and the absolute value of negative cohesion. All analyses were conducted in the R environment (v. 4.3.3).

### 2.5 Analysis of habitat specialists and generalists

To assess microbial habitat specialization, we calculated the niche width of each OTU based on Levins’ niche breadth index, which quantifies the range of environmental conditions that species can tolerate (Levins, 1968). The OTUs with high niche breadth values were classified as belonging to generalists and those with low values as belonging to specialists. To statistically assess the significance of niche width, we generated null distributions by conducting 1,000 permutations of the OTU table using the *permatswap* function (vegan package) in R (v. 4.3.3). In each iteration, the OTU matrix was randomized once, and niche width was recalculated based on the permuted matrix. The observed niche width values were then compared to the corresponding null distributions to evaluate their deviation from random expectations. The OTUs with niche width values exceeding the 95% confidence interval of the null distribution were assigned as belonging to generalists, whereas those below the lower bound were identified as belonging to specialists. The OTUs falling within the 95% interval were considered neutral (Du et al., 2025). All computations were conducted in the R environment (v. 4.3.3), using the packages *vegan*, *permute*, and *ggplot2*. To assess differences in community structure, principal coordinates analysis (PCoA) based on Bray–Curtis dissimilarity was employed to examine the distribution patterns of specialist and generalist taxa (Du et al., 2025).

### 2.6 Functional prediction of bacterial communities

*PICRUSt2* was used to infer predicted bacterial functional potential from 16S rRNA amplicon profiles (Yang et al., 2023; Douglas et al., 2020). Functional annotations were assigned as KEGG orthologs and further mapped to hierarchical KEGG pathways. The relative abundances of predicted pathways were normalized and compared between HS and LS groups to identify potential differences in functional profiles. Because *PICRUSt2* infers functional potential from marker-gene profiles, these results were interpreted as predictions rather than direct measurements of gene abundance, transcription, protein expression or metabolic activity. Therefore, pathway-level differences were used to generate hypotheses about microbial functional responses to saline-alkali conditions rather than to infer realized microbial functions. All functional analyses and visualizations were conducted in the R environment (v. 4.3.3), using the *phyloseq*, *vegan*, and *ggplot2* packages.

### 2.7 Statistical analysis

All statistical analyses were conducted in R v. 4.3.3 unless otherwise stated. Soil physicochemical variables were first summarized for each sampling site using the three plot-level composite samples. Values in Supplementary Table S1 are presented as mean ± standard deviation. Differences among sampling sites were evaluated by one-way analysis of variance followed by Duncan’s multiple range test at *p* < 0.05. For comparisons among EC-defined LS, MS and HS groups, site-level means were used as independent observations (LS, *n* = 3 sites; MS, *n* = 4 sites; HS, *n* = 3 sites). Differences in soil physicochemical variables among groups were tested using one-way analysis of variance followed by *Tukey’s HSD* test. When the assumptions of normality or homogeneity of variance were not met, Kruskal–Wallis tests followed by Dunn’s *post hoc* comparisons were used.

**Figure 2:**
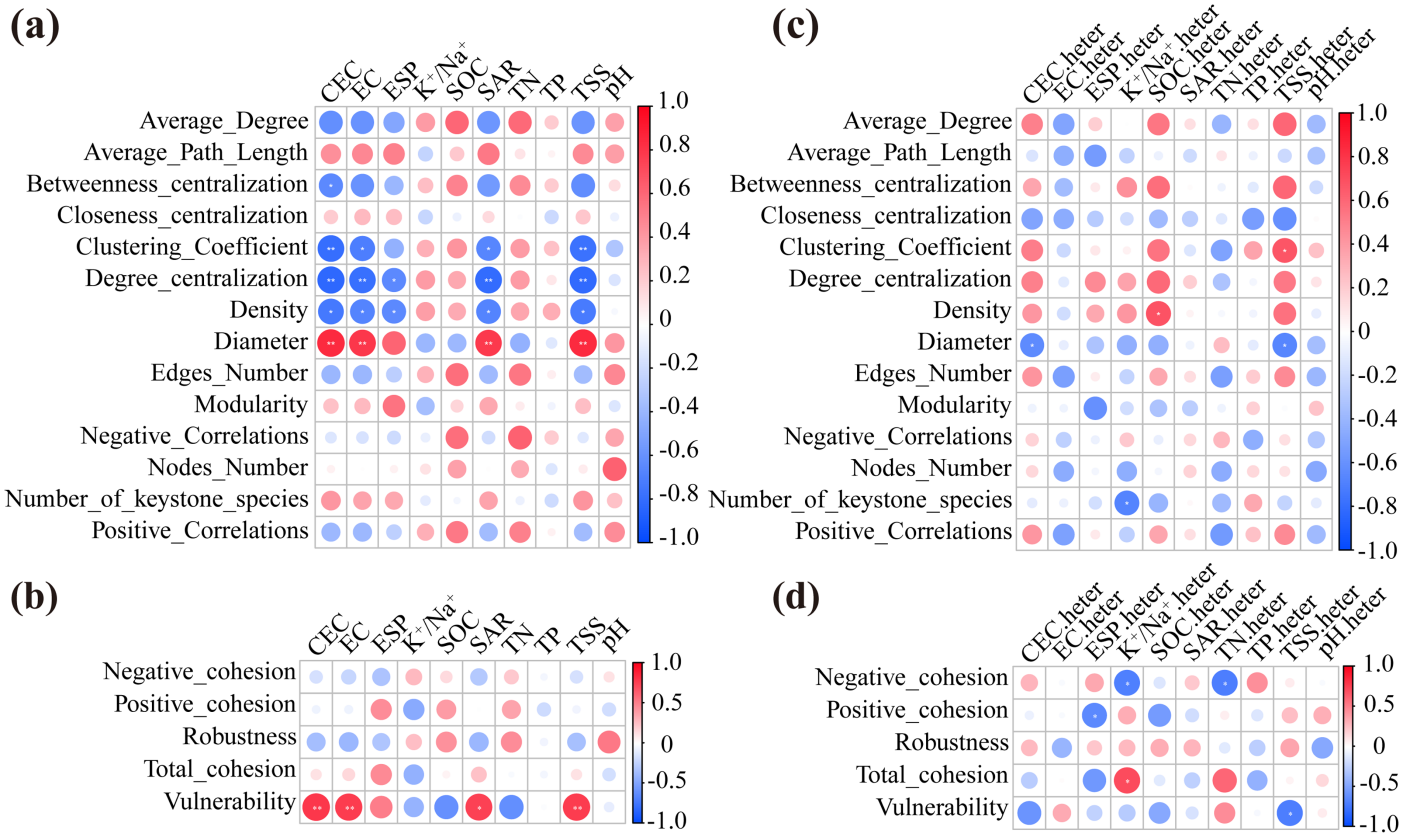
Relationships between environmental variables, environmental heterogeneity, and indicators of soil microbial network complexity and stability. **(a)** Show the relationships between environmental variables and microbial network complexity, **(b)** environmental variables and network stability, **(c)** heterogeneity of soil properties and network complexity, **(d)** heterogeneity of soil properties and network stability. The size of each circle represents the magnitude of the correlation coefficient. Red and blue circles indicate positive and negative correlations, respectively. CEC, cation exchange capacity; EC, electrical conductivity; ESP, exchangeable sodium percentage; TSS, total soluble salts; K^+^/Na^+^, potassium-to-sodium ratio; SAR, sodium adsorption ratio; SOC, soil organic carbon; TN, total nitrogen; TP, total phosphorus; pH, soil pH. “Heter” indicates the spatial heterogeneity of the corresponding soil variable. *p < 0.05; **p < 0.01.

Environmental heterogeneity was calculated separately for each soil physicochemical variable at each sampling site using the three plot-level composite samples. Specifically, heterogeneity was expressed as the coefficient of variation (CV), calculated as the standard deviation divided by the mean value of the corresponding variable. These site-level heterogeneity values were used in the correlation analyses shown in Figures 2c,d.

Associations between soil physicochemical variables, environmental heterogeneity and microbial network topological properties were evaluated using Spearman rank correlation analysis. Network topological properties were calculated at the site level and matched with the corresponding site-level soil variables or heterogeneity values.

For specialist and generalist analyses, Chao1 and Shannon indices were compared among niche-breadth categories using Kruskal–Wallis tests followed by Dunn’s post hoc comparisons. Spearman rank correlation analysis was used to evaluate relationships between alpha-diversity indices and soil physicochemical variables. PCoA was performed based on *Bray–Curtis* dissimilarity. Differences in predicted KEGG pathway proportions between HS and LS groups were evaluated using Welch’s *t*-test, with effect sizes reported as differences in mean proportions and 95% confidence intervals. *p*-values were corrected for multiple testing using the false-discovery-rate method.

## 3 Results

### 3.1 Changes in soil variables under heterogeneous saline-alkali conditions

Soil color and texture varied substantially across the sampling sites, indicating marked heterogeneity in soil background conditions across the investigated regions (Figure 1b). Soil electrical conductivity (EC), used as the primary indicator of saline-alkali intensity, differed significantly among the HS, MS and LS groups (Figure 1c). Several other physicochemical properties also varied across the EC-defined saline-alkali intensity gradient, including pH, total soluble salts (TSS), total nitrogen (TN), total phosphorus (TP) and soil organic carbon (SOC). With increasing EC, TN, TP and SOC showed significant negative correlations, whereas soil pH and TSS showed positive correlations. The EC-based grouping was further supported by Supplementary Table S1, as HS soils generally showed higher TSS, SAR and ESP values and lower K^+^/Na^+^ ratios than LS soils, indicating stronger soluble salt accumulation, sodicity and ionic imbalance. However, pH and sodicity-related indicators did not vary monotonically across all LS, MS and HS sites, reflecting regional soil heterogeneity. These patterns indicate that higher saline-alkali intensity was associated with reduced nutrient availability and altered soil chemical conditions across the sampled sites. Together, these physicochemical gradients provided the environmental basis for evaluating microbial community, network and niche-breadth patterns across EC defined saline-alkali intensity categories.

### 3.2 Effects of saline-alkali on soil microbial network complexity and stability

Among the 14 topological features of microbial networks, several indices related to network complexity were significantly associated with saline-alkali conditions. Clustering coefficient, degree centralization and network density showed significant negative correlations with saline-alkali indicators, indicating that microbial networks became less tightly connected as saline-alkali intensity increased (Figure 2a). In contrast, CEC, EC, SAR and TSS were significantly positively correlated with network diameter and vulnerability, suggesting that stronger saline-alkali conditions were associated with more dispersed network structures and greater sensitivity to disturbance (Figure 2b). Spatial heterogeneity in soil properties was also linked to microbial network topology. The heterogeneity of CEC and TSS was significantly negatively correlated with network diameter, whereas heterogeneity in the K^+^/Na^+^ ratio was negatively correlated with the number of keystone species (Figure 2d). In addition, SOC heterogeneity was significantly negatively associated with network density, and TSS heterogeneity was negatively associated with the clustering coefficient (Figure 2c). The heterogeneity of ESP, K^+^/Na^+^ and TSS showed significant negative correlations with positive cohesion, negative cohesion and vulnerability, respectively, whereas no significant correlations were detected for the other variables (Figure 2d). Together, these results indicate that higher saline-alkali intensity and spatial heterogeneity in key soil properties were associated with reduced microbial network complexity and altered network stability. We next examined whether generalist and specialist taxa differed in their diversity and community composition across the saline-alkali gradient.

### 3.3 Generalists and specialists display contrasting diversity and compositional shifts in response to saline-alkali

**Figure 3:**
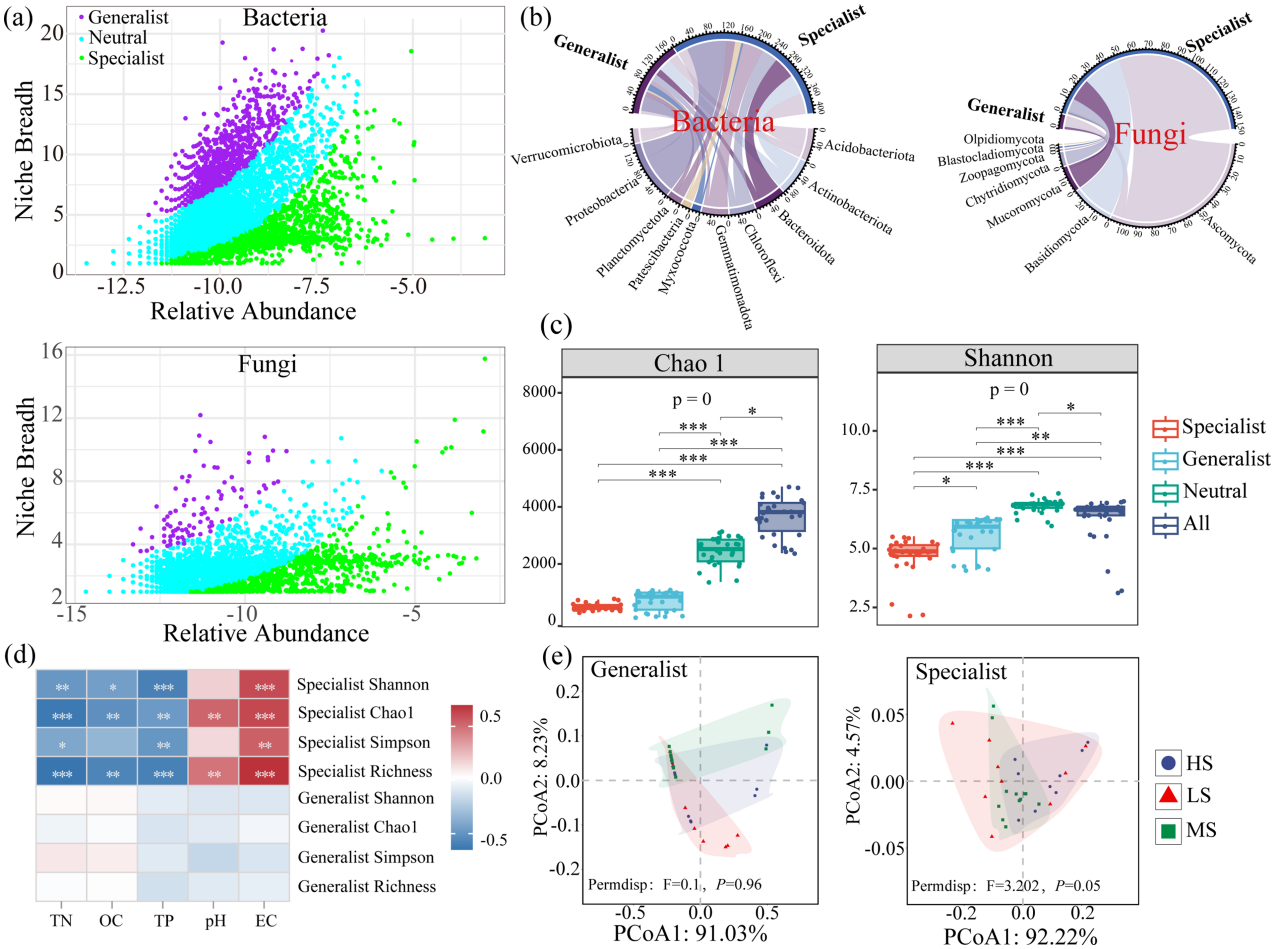
Diversity and community structure of specialist and generalist taxa in saline-alkaline soils. **(a)** Identification of bacterial and fungal specialists and generalists. **(b)** Community composition of specialists and generalists at the phylum level. **(c)** Chao1 and Shannon indices of specialists and generalists. **(d)** Spearman correlation analysis between environmental variables and *α*-diversity indices. **(e)** Principal coordinates analysis (PCoA) of specialists and generalists. ***, *p* < 0.001; **, *p* < 0.01; *, *p* < 0.05.

The soil microbial communities from the 30 samples were characterized using bacterial 16S rRNA and fungal ITS amplicon sequencing. After quality filtering and OTU selection, 16,910 bacterial OTUs and 4,454 fungal OTUs were retained for downstream analyses. Based on niche breadth classification, 1,766 bacterial OTUs (10.4%) were identified as specialists and 1,364 OTUs (8.1%) as generalists. In the fungal community, 841 OTUs (18.9%) were classified as specialists and 99 OTUs (2.2%) as generalists (Figure 3a). At the phylum level, bacterial specialists were mainly affiliated with Proteobacteria, Bacteroidota and Gemmatimonadota, whereas bacterial generalists were dominated by Proteobacteria, Actinobacteriota and Acidobacteriota. Fungal specialists mainly comprised Ascomycota, Basidiomycota, Mucoromycota and Chytridiomycota, whereas fungal generalists were mainly represented by Ascomycota, Mucoromycota, and Basidiomycota (Figure 3b). The *α*-diversity of bacterial generalists was higher than that of bacterial specialists (Figure 3c). Correlation analysis between environmental variables and microbial diversity showed that the α-diversity of bacterial specialists was significantly positively correlated with EC, whereas no significant correlations were observed for bacterial generalists (Figure 3d). This pattern was not detected in fungal communities, as neither fungal specialists nor fungal generalists showed significant associations between α-diversity and soil physicochemical properties (Figure 3d). PCoA further showed that bacterial specialists exhibited compositional shifts across saline-alkali gradients (*F* = 3.202, *p* = 0.05), whereas bacterial generalists showed no significant separation among groups (*F* = 0.1, *p* = 0.96) (Figure 3e). These contrasting patterns indicate that bacterial specialists contributed more strongly than generalists to community differentiation along the saline-alkali gradient.

### 3.4 Effects of soil saline-alkali on fungal-bacterial co-occurrence network complexity and key taxa composition

**Figure 4:**
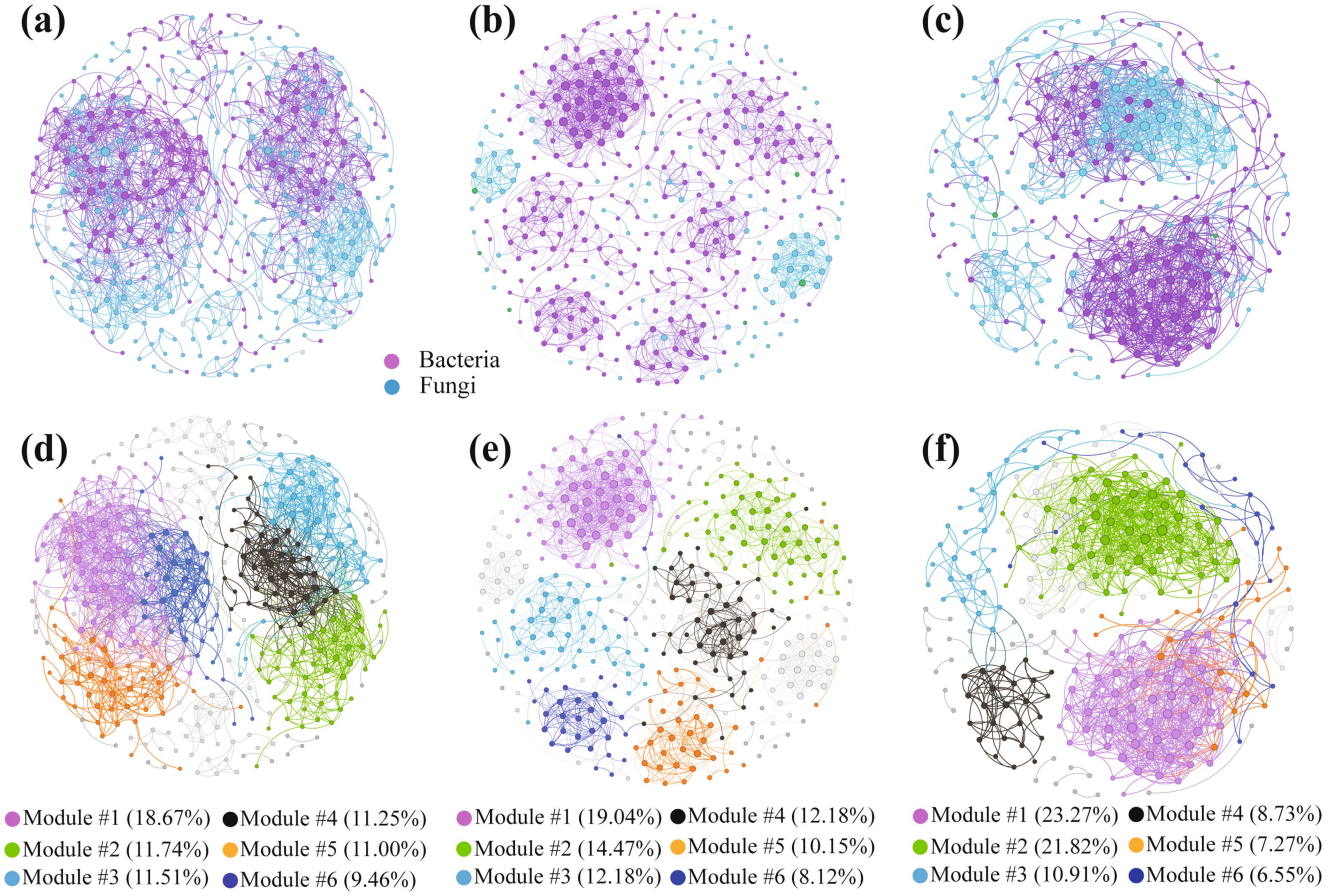
Fungal-bacterial co-occurrence networks across soils with different salinity levels. Node size is proportional to node degree, and edges represent strong (*r* > 0.8) and significant correlations (*p* < 0.01). **(a–c)** show fungal-bacterial OTUs co-occurrence networks in HS, MS, and LS groups, respectively; **(d–f)** depict the corresponding modularity analyses for the co-occurrence networks in the HS, MS, and LS groups.

To explore fungal-bacterial co-occurrence patterns across different saline-alkali levels, three group-level networks were constructed for HS, MS and LS soils (Figures 4a–c). The HS network contained 391 nodes and 1,803 edges, the MS network contained 487 nodes and 4,777 edges, and the LS network contained 286 nodes and 2,362 edges. Compared with the LS network, the HS network contained more nodes but fewer edges, indicating that high saline-alkali conditions were associated with a less connected fungal-bacterial co-occurrence structure (Supplementary Table S2). Modularity analysis identified six major modules in each network, accounting for 73.63, 76.14, and 78.55% of the total nodes in the HS, MS and LS networks, respectively (Figures 4d–f). These results indicate that modular organization was retained across saline-alkali levels, although the distribution of microbial taxa among modules varied among groups. Taxonomic composition within the networks showed distinct bacterial and fungal patterns. Proteobacteria was the predominant bacterial phylum in all networks, accounting for 13.55, 15.81, and 17.48% of nodes in the HS, MS and LS networks, respectively. Its relative representation decreased with increasing saline-alkali intensity. In contrast, Ascomycota was the predominant fungal phylum across the networks, accounting for 25.83, 18.28, and 23.78% of nodes in the HS, MS and LS networks, respectively, with no clear directional change across saline-alkali levels.

### 3.5 Comparative analysis of microbial functional profiles between high and low saline-alkali

**Figure 5:**
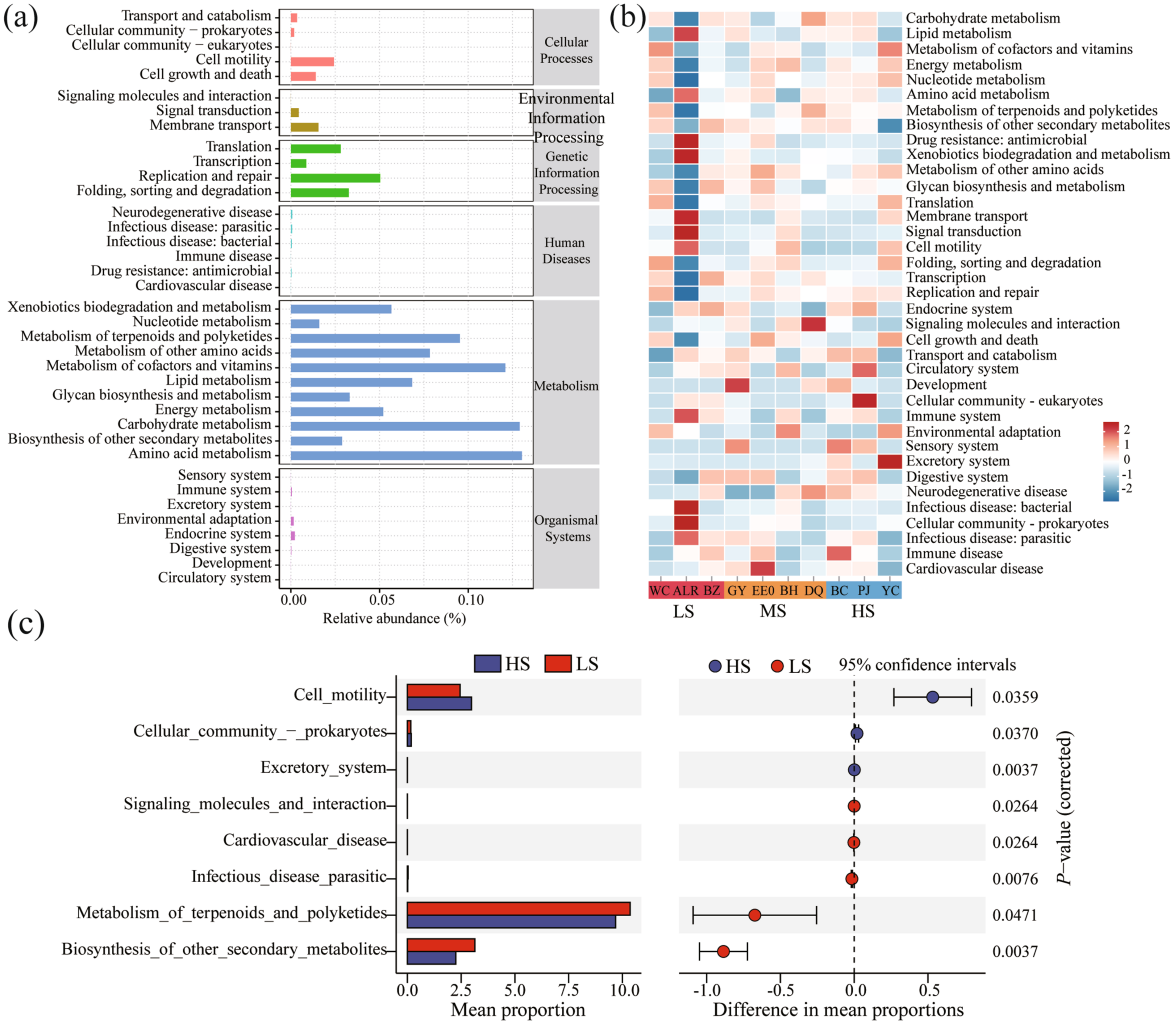
Functional profiling of predicted microbial KEGG pathways under different saline-alkali conditions. **(a)** Stacked bar plots showing the relative abundance of predicted KEGG level 2 pathways in HS and LS groups. Functional categories are colored based on KEGG level 1 classification. **(b)** Heatmap visualization of KEGG pathway profiles across multiple samples. Functional clustering reveals variation and patterns among treatment groups. **(c)** Differential abundance analysis indicating the difference in mean proportions between HS and LS groups for each pathway, along with 95% confidence intervals.

To explore potential shifts in bacterial functional profiles under different saline-alkali conditions, we predicted KEGG pathway profiles from 16S rRNA amplicon data using *PICRUSt2*. Both HS and LS groups were dominated by predicted metabolism-related pathways, particularly carbohydrate metabolism, amino acid metabolism, and metabolism of cofactors and vitamins (Figure 5a). Other KEGG level-2 categories were detected at lower relative abundances, indicating that the predicted pathway profiles were broadly distributed across multiple functional classes. Heatmap analysis showed that predicted pathway profiles differed among samples, and samples with similar saline-alkali levels tended to cluster more closely (Figure 5b). To further compare predicted pathway composition, representative HS and LS samples with contrasting saline-alkali levels were selected for pathway abundance comparison (Figure 5c). The representative LS sample showed higher predicted relative abundances of pathways related to terpenoid and polyketide metabolism and biosynthesis of other secondary metabolites. In contrast, the representative HS sample showed higher predicted relative abundance of cell motility-related pathways. Overall, these results suggest that saline-alkali conditions were associated with shifts in predicted bacterial functional potential, with possible enrichment of stress-survival-related pathways under high saline-alkali conditions. Because these results were inferred from 16S rRNA marker-gene data, they should be interpreted as predicted functional potential rather than direct evidence of gene abundance, expression or metabolic activity.

## 4 Discussion

### 4.1 Soil saline-alkali alters soil physicochemical properties and nutrient availability

Soil saline-alkali is known to negatively affect soil structure, nutrient dynamics, and microbial diversity (Wang et al., 2021). Based on our results, we confirm that increasing saline-alkali, as indicated by high pH and TSS levels, is associated with significant reductions in TN, TP, and SOC contents. These findings support our initial hypothesis that salt-alkaline stress lowers the soil nutrient status. These results highlight how saline-alkali induced physicochemical changes in soil properties can impair soil fertility and microbial habitat quality. Under saline-alkali conditions, osmotic stress and ion toxicity disrupt nutrient retention and biochemical cycling, thereby inhibiting the accumulation of essential macronutrients such as nitrogen and phosphorus (Wang et al., 2025). This pattern may be associated with sodium-induced soil particle dispersion and reduced microbial decomposition of organic matter (Xing et al., 2024). Similar declines in soil organic carbon and nitrogen under increasing saline-alkali conditions have also been reported previously (Hassani et al., 2024). More broadly, recent evidence from Chinese dryland agroecosystems indicates that SOC sequestration can be closely linked to microbial carbon-use efficiency and microbial-derived C formation (Liu Z. et al., 2025). In this context, the SOC decline observed along the saline-alkali gradient may indicate reduced nutrient availability and potential constraints on microbial-mediated carbon stabilization. This proposed mechanism should be tested in future work by directly quantifying microbial carbon-use efficiency, microbial residues and carbon-cycling functional genes. These patterns suggest that saline-alkali functions not only as a direct osmotic constraint but also as a key environmental filter by limiting resource availability for microbial metabolism (Li M. et al., 2024). In contrast to previous studies that focused on sodium concentration or exchangeable cations, in this study, the combined impacts of EC, pH, and TSS in shaping nutrient dynamics under heterogeneous saline conditions are highlighted. In summary, regulating saline-alkali accumulation and stabilizing soil pH can help reduce nutrient loss and enhance soil ecological functioning, providing new insights into the mechanisms of saline-alkali driven soil degradation.

### 4.2 Saline-alkali weakens the complexity and stability of soil microbial networks

Microbial network topological properties offer valuable insights into community structure and function. They help identify keystone species, assess ecosystem stability, define microbial functional zones, and predict community dynamics (Kajihara and Hynson, 2024). The results of the present study demonstrate that saline-alkali stress negatively affects key features of soil microbial networks, particularly the clustering coefficient, degree centralization, and density, which are critical indicators of network complexity (Figure 2). These findings are consistent with previous studies showing that salinization and other environmental stresses are associated with reduced microbial network complexity and stability (He et al., 2025; Luo et al., 2025; Zhang et al., 2024). Conversely, indicators associated with saline-alkali, such as EC, CEC, SAR, and TSS, were positively associated with network diameter and vulnerability, suggesting an expansion of interaction distance and an increased sensitivity of networks to disturbance under saline-alkali conditions (Figures 2a,b). One explanation for these results is that higher ionic strength and osmotic stress may constrain microbial growth, resource acquisition and dispersal, thereby limiting the formation of stable and compact microbial sub-networks (He et al., 2025). These findings are consistent with previous studies showing that salinity, aridity and other environmental stresses are associated with reduced microbial network complexity and stability (He et al., 2025; Luo et al., 2025; Wang et al., 2023). Increases in network diameter and vulnerability may indicate a shift toward more loosely connected communities that are more susceptible to environmental disturbance and species loss (Chen et al., 2023; Kajihara and Hynson, 2024).

An important result of our study is that the heterogeneity of specific saline-alkali factors, such as ESP, K^+^/Na^+^, and TSS, was negatively associated with both positive and negative cohesion and vulnerability. This suggests that spatial heterogeneity in saline-alkali-related soil properties may be linked to less cohesive microbial association patterns. Similar reductions in microbial network complexity and stability under saline-alkali stress have also been reported previously (Chen et al., 2024). However, our results extend this view by demonstrating that ionic imbalance can further destabilize microbial networks. Specifically, the significant negative correlation between K^+^/Na^+^ heterogeneity and the number of keystone species indicates that such imbalance reduces the presence or persistence of key microbial taxa essential for maintaining network structure. In addition, significant negative correlations between the heterogeneity of SOC and TSS with density and clustering coefficient, respectively, indicate that uneven distribution of organic carbon and saline-alkali weakens microbial aggregation and cooperation. These findings highlight the dual impact of saline-alkali: it acts not only as a direct stressor that reduces microbial complexity but also diminishes the stabilizing influence of environmental heterogeneity on shaping microbial community resilience (Luo et al., 2025). In summary, saline-alkali stress undermines both the structural integrity and ecological stability of soil microbial networks by decreasing interaction density and increasing network vulnerability. These destabilizing effects are intensified by spatial heterogeneity in key soil properties, underscoring the importance of addressing both saline-alkali levels and their distribution when assessing microbial responses to soil degradation in saline environments.

### 4.3 Specialists show stronger bacterial community responses under saline-alkali conditions

We observed distinct responses of generalist and specialist bacterial taxa to saline-alkali stress. Bacterial generalists showed higher *α*-diversity levels than specialists, indicating broader niche adaptability and consequently greater ecological tolerance. This result is consistent with previous studies demonstrating that generalists often thrive across a wide range of environments owing to their metabolic flexibility and capacity to utilize different resources (Schaerer et al., 2023). However, our results also revealed that bacterial specialists, rather than generalists, had a significant positive correlation between α-diversity and saline-alkali, indicating that certain specialists are better suited to saline-alkali conditions. This indicates that environmental heterogeneity under stress further destabilizes microbial interactions. In turn, this may contribute to functional compensation within the microbial community, potentially supporting plant resilience under adverse conditions (Du et al., 2025).

Notably, this saline-alkali diversity relationship was only observed among bacterial specialists. No significant associations were found for bacterial generalists or fungal groups, indicating a domain-specific adaptive strategy. Bacterial specialists may possess unique genetic or physiological traits, such as osmoprotectant production or saline-alkali tolerant transport systems, that enhance their survival under high saline-alkali, although this interpretation requires genomic, culture-based or experimental validation. By contrast, the absence of clear correlations between fungal diversity and soil properties may reflect greater compositional stability or stress tolerance of fungal communities under the sampled saline-alkali gradient, consistent with previous evidence from stress-gradient studies (Winterfeldt et al., 2024). Moreover, while the composition of bacterial specialists changed significantly along the saline-alkali gradient, generalists remained relatively unchanged, reflecting a more stable response to environmental stress. This contrast underscores the greater sensitivity of specialists to environmental gradients, most likely because of their narrower niches and, consequently, reduced ecological plasticity. Previous studies have suggested that shifts in the community composition of specialists may arise from habitat filtering, a process where environmental stress favors specific taxa and reshapes the overall community structure (Philippot et al., 2024).

The taxonomic composition of specialists and generalists showed clear distinctions. Bacterial specialists were primarily affiliated with Proteobacteria, Bacteroidota, and Gemmatimonadota, often associated with organic matter decomposition and nutrient cycling under stress conditions. In contrast, generalists were dominated by Proteobacteria, Actinobacteriota, and Acidobacteriota, reflecting their broader functional roles and tolerance across diverse environments. For fungi, both specialists and generalists were primarily composed of Ascomycota and Mucoromycota, likely due to their shared ecological functions. However, a higher abundance of Chytridiomycota and Cercozoa in specialists suggests niche-specific adaptations to saline-alkali induced constraints (Du et al., 2025). In summary, our findings reveal distinct diversity and composition in the responses of generalists and specialists to saline-alkali. While generalists showed relatively stable patterns, specialists showed stronger diversity and compositional associations with saline-alkali conditions, which highlights their important role in shaping microbial communities in saline-alkali soils.

### 4.4 Soil saline-alkali reduces fungal-bacterial network complexity and alters key taxonomic representation

Our findings show that fungal-bacterial co-occurrence network structure differed across saline-alkali levels. Under HS, the number of network nodes increased, indicating greater taxonomic richness. However, this was accompanied by a substantial reduction in the number of edges, suggesting weakened co-occurrence patterns. This decoupling implies that although more microbial taxa were present, their interactions were diminished, likely due to disrupted ecological relationships or increased niche differentiation. The observed decrease in edge density implies reduced connectivity among taxa, which may compromise overall network robustness and ecological coordination. Such decoupling between taxonomic richness (nodes) and interaction intensity (edges) under saline-alkali stress may reflect fragmented microbial communities. Similar patterns have been reported for stress-affected ecosystems, where environmental filtering allows more taxa to persist but limits their ability to form stable associations (Zhang et al., 2024; Chen et al., 2017). These results suggest that saline-alkali enhances microbial heterogeneity while impairing mutualistic or competitive linkages essential for complex network organization.

The modular structure of the networks remained relatively stable, with six dominant modules accounting for over 70% of the total nodes across all saline-alkali levels. However, slight decreases in modular proportions from LS to HS suggest that increased saline-alkali may limit microbial compartmentalization, potentially reducing the system’s resilience to environmental disturbances. Taxonomic analysis further revealed distinct responses between bacteria and fungi. Proteobacteria, the dominant bacterial phylum, showed a gradual decline in relative abundance with increasing saline-alkali, indicating its sensitivity to ionic stress (Luo et al., 2025). In contrast, in another study, Ascomycota remained the predominant fungal phylum across all networks, with no significant change in relative abundance (Wu et al., 2024). These findings highlight domain-specific responses of microbial taxa to environmental gradients: bacterial communities appear more susceptible to structural shifts, whereas fungal communities exhibit greater compositional stability (de Vries et al., 2018). Overall, fungal-bacterial co-occurrence networks varied across saline-alkali levels, with higher taxonomic richness but lower connectivity under HS conditions. The shift in bacterial dominance and the stability of fungal representation underscore the importance of considering both interaction networks and taxonomic composition when evaluating microbial resilience to soil saline-alkali.

### 4.5 Saline-alkali stress induces functional shifts in soil microbial communities

The predicted functional profiles suggested that microbial communities differed between high and low saline-alkali soils. Both HS and LS groups were dominated by core metabolism-related pathways, including carbohydrate metabolism, amino acid metabolism and the metabolism of cofactors and vitamins. This pattern is expected because these pathways represent conserved microbial functions that support growth and maintenance across soil environments (Dai et al., 2021; Zhang et al., 2022). However, predicted pathway differences between HS and LS samples indicate that saline-alkali conditions may be associated with shifts in microbial functional potential. Because microbial carbon-use efficiency and microbial-derived carbon formation are increasingly recognized as important controls on SOC sequestration in dryland agroecosystems (Liu Z. et al., 2025), these predicted functional differences may provide a useful hypothesis for linking saline-alkali stress, microbial community organization and soil carbon loss. Because *PICRUSt2* infers functional potential from 16S rRNA phylogenetic information, future metagenomic, metatranscriptomic or targeted *qPCR* analyses are needed to directly quantify the corresponding functional genes and their expression (Yang et al., 2023; Douglas et al., 2020).

The LS sample showed higher predicted representation of pathways related to terpenoid and polyketide metabolism and the biosynthesis of other secondary metabolites. These pathways are often linked to microbial chemical communication, antagonism and interaction with hosts or neighboring microorganisms (Jacoby et al., 2021). In contrast, the HS sample showed higher predicted representation of cell motility-related pathways, which may reflect the importance of movement, chemotaxis or microsite tracking under stronger saline-alkali constraints. Under stressful soil conditions, microbial taxa capable of locating favorable microhabitats or responding rapidly to chemical gradients may have a selective advantage (Zheng et al., 2024; Trivedi et al., 2020).

Overall, the predicted functional results suggest a potential shift from interaction-related and secondary-metabolism-associated functions under low saline-alkali conditions toward stress-survival-related functional potential under high saline-alkali conditions. This interpretation is consistent with the observed reduction in network connectivity, because weaker association structure may coincide with reduced representation of functions related to microbial communication and ecological interaction. However, these results remain hypothesis-generating. Future shotgun metagenomic, metatranscriptomic or culture-based studies are needed to verify whether the predicted functional differences correspond to actual gene abundance, gene expression or microbial activity.

This study has several limitations. First, although the sampling sites covered a broad geographic range, the number of sites was limited, which restricts the ability to separate saline-alkali effects from other regional environmental drivers. Second, site-level climatic conditions, vegetation type, land-use history and soil type were not incorporated into the statistical framework. These factors may partially co-vary with saline-alkali conditions and contribute to microbial community variation. Third, co-occurrence network analysis identifies statistical associations among taxa but cannot directly confirm ecological interactions. Finally, *PICRUSt2* provides predicted functional potential rather than direct evidence of functional genes, transcripts or metabolic activity. Therefore, the observed microbial patterns should be interpreted as associations with measured saline-alkali soil conditions, and future studies should combine denser spatial sampling, environmental metadata, shotgun metagenomics and controlled experiments to test the mechanisms proposed here.

## 5 Conclusion

This study shows that bacterial and fungal communities, co-occurrence networks, niche breadth patterns and predicted functional potential vary along saline-alkali gradients in Chinese soils. Higher saline-alkali intensity was associated with reduced nutrient availability, lower network complexity and stronger responses of bacterial specialists, whereas fungal communities showed comparatively stable patterns. Predicted functional profiles suggested potential shifts toward stress-survival-related functions under high saline-alkali conditions. These findings provide microbial ecological indicators for saline-alkali soil assessment, but their functional roles require validation through metagenomic, culture-based and experimental approaches.

## Data Availability

The raw sequence data have been deposited in the Genome Sequence Archive at the National Genomics Data Center, China National Center for Bioinformation/Beijing Institute of Genomics, Chinese Academy of Sciences, under accession number CRA034051, and are publicly accessible at: https://ngdc.cncb.ac.cn/gsa.
