# Supplementary material for: Saline-alkali gradients reshape soil microbial network complexity and niche breadth
Source: Front Microbiol. 2026 Jun 29;17:1886660. doi: 10.3389/fmicb.2026.1886660 (PMC13357909; doi:10.3389/fmicb.2026.1886660)
Supplement: Supplementary file 1 [file Data_Sheet_1.DOCX]

**Table S1** Physical and chemical properties of soil at sampling points

| **Groups** | Region | Latitude/Longitude | TN (g kg^-1^) | SOC (g kg^-1^) | TP (g kg^-1^) | pH | EC (dS·m^-1^) | SAR | ESP (%) | CEC | TSS (g/kg) | K^+^/Na^+^ |
| --- | --- | --- | --- | --- | --- | --- | --- | --- | --- | --- | --- | --- |
| **LS** | ALR | 40°31′N/81°22′E | 0.57±0.01g | 5.62±0.18i | 0.88±0.01c | 8.07±0.03f | 0.18±0.00h | 28.25±0.40i | 12.72±0.24i | 934.79±14.87g | 0.76±0.01i | 1.53±0.04b |
|  | WC | 20°02′N/110°37′E | 1.01±0.02c | 10.11±0.11c | 0.20±0.00j | 5.66±0.05h | 0.15±0.00i | 274.30±3.59f | 77.79±0.30b | 656.09±6.40h | 0.64±0.01j | 0.03±0.01f |
|  | BZ | 38°03′N/117°36′E | 1.53±0.05b | 14.27±0.23b | 1.28±0.01a | 8.15±0.03e | 0.18±0.00h | 78.13±0.50h | 30.78±0.15g | 1026.89±13.00g | 0.85±0.02h | 0.30±0.02d |
| **MS** | GY | 36°08′N/106°02′E | 0.94±0.01d | 8.21±0.16f | 0.67±0.01f | 8.08±0.02f | 0.24±0.01g | 40.56±0.33i | 14.43±0.18h | 1326.87±16.65f | 0.92±0.02g | 2.17±0.04a |
|  | EE0 | 40°28′N/110°01′E | 0.75±0.01e | 9.32±0.22d | 0.86±0.02d | 8.94±0.03c | 0.28±0.00f | 311.90±2.49e | 66.06±0.32d | 1632.93±7.76e | 1.23±0.02e | 0.12±0.00e |
|  | BH | 21°27′N/109°13′E | 0.76±0.01e | 7.70±0.08g | 0.78±0.02e | 4.52±0.04i | 0.40±0.01e | 241.27±1.35g | 58.83±0.32e | 1557.58±23.15e | 1.01±0.02f | 0.12±0.00e |
|  | DQ | 46°35′N/125°10′E | 2.19±0.02a | 20.96±0.27a | 0.59±0.01g | 9.12±0.05b | 0.50±0.00d | 423.27±10.33d | 58.84±0.42e | 3902.76±80.76d | 2.58±0.04d | 0.32±0.00d |
| **HS** | BC | 45°50′N/123°03′E | 0.65±0.01f | 6.36±0.03h | 0.30±0.01i | 9.97±0.04a | 0.93±0.02c | 673.70±20.73c | 54.16±0.85f | 12481.57±144.68b | 5.37±0.10c | 0.43±0.01c |
|  | PJ | 41°0′N/122°04′E | 0.96±0.02d | 8.78±0.05e | 0.51±0.00h | 7.54±0.03g | 2.63±0.01b | 1047.20±6.21b | 73.98±0.22c | 12347.66±36.48c | 6.21±0.02b | 0.03±0.00f |
|  | YC | 32°59′N/120°49′E | 0.46±0.02h | 5.82±0.07i | 0.89±0.01b | 8.71±0.02d | 6.03±0.05a | 3307.65±21.25a | 89.90±0.07a | 30644.34±117.05a | 14.26±0.02a | 0.02±0.00f |

Note: Values are presented as mean ± standard deviation. Different lowercase letters within a column indicate significant differences among sampling points at *p* < 0.05. CEC, cation exchange capacity; EC, electrical conductivity; ESP, exchangeable sodium percentage; TSS, total soluble salts; K^+^/Na^+^, sodium to potassium ratio; SAR, sodium adsorption ratio; SOC, soil organic carbon; TN, total nitrogen; TP, total phosphorus; pH, soil pH.

**Table S2** Description of Topological features of soil microbial networks in major saline-alkali regions of China

| Topological feature | Description |
| --- | --- |
| Nodes_Number | Number of OTUs in the network |
| Edges_Number | Number of potential associations between OTUs |
| Diameter | The greatest distance among the nodes in the network |
| Density | The ratio of the number of edges and the number of possible edges |
| Modularity | Modular scores of a given division of a graph into different modules |
| Average_Degree | The average connections of each node with another particular node |
| Average_Path_Length | The average network distance between all pairs of nodes |
| Clustering_Coefficient | The degree to which the nodes tend to cluster together |
| Degree centralization | Creating a graph level centralization measure from the centrality scores of the nodes |
| Betweenness centralization | The mean of betweenness centrality values for all nodes |
| Closeness centralization | The mean of closeness centrality values for all nodes |
| Number of keystone species | Species that play a key role in maintaining network biodiversity, structure, function, and stability |
| Positive_Correlations | The Positively correlated species within the network |
| Negative_Correlations | Species showing negative correlations in the network |
| Positive_Cohesion | The mutually beneficial interactions among microorganisms |
| Negative_Cohesion | The competitive or antagonistic interactions among microbial populations |
| Total_Cohesion | Total cohesion is defined as the combined absolute values of positive and negative cohesion |
| Robustness | The network's resistance to node loss following random or targeted node removal |
| Vulnerability | The Network vulnerability is defined by the maximum vulnerability of its nodes |

**Table S3** Topological properties of soil microbial networks across the ten sites

| Taxon | WC | ALR | BZ | GY | EE0 | BH | DQ | BC | PJ | YC |
| --- | --- | --- | --- | --- | --- | --- | --- | --- | --- | --- |
| Nodes_Number | 241 | 225 | 280 | 446 | 470 | 227 | 467 | 373 | 388 | 316 |
| Edges_Number | 1398 | 1486 | 2292 | 4048 | 4435 | 661 | 4499 | 1663 | 1767 | 826 |
| Positive_Correlations | 1125 | 1252 | 1777 | 3831 | 4246 | 590 | 4156 | 1391 | 1437 | 641 |
| Negative_Correlations | 273 | 234 | 515 | 217 | 189 | 71 | 343 | 272 | 330 | 185 |
| Average_Degree | 11.60 | 13.21 | 16.37 | 18.15 | 18.87 | 5.82 | 19.27 | 8.92 | 9.11 | 5.23 |
| Average_Path_Length | 4.35 | 3.83 | 4.22 | 4.64 | 5.03 | 4.13 | 4.79 | 4.24 | 4.37 | 5.07 |
| Diameter | 13.00 | 13.11 | 13.07 | 13.77 | 16.49 | 13.77 | 13.82 | 17.17 | 18.15 | 19.09 |
| Density | 0.05 | 0.06 | 0.06 | 0.04 | 0.04 | 0.03 | 0.04 | 0.02 | 0.02 | 0.02 |
| Clustering_Coefficient | 0.60 | 0.60 | 0.60 | 0.66 | 0.66 | 0.66 | 0.66 | 0.47 | 0.47 | 0.47 |
| Modularity | 0.67 | 0.48 | 0.60 | 0.71 | 0.76 | 0.77 | 0.73 | 0.63 | 0.62 | 0.77 |
| Degree centralization | 0.12 | 0.15 | 0.12 | 0.10 | 0.09 | 0.08 | 0.11 | 0.07 | 0.08 | 0.03 |
| Betweenness centralization | 0.13 | 0.11 | 0.14 | 0.11 | 0.13 | 0.04 | 0.12 | 0.06 | 0.06 | 0.05 |
| Closeness centralization | 0.00 | 0.00 | 0.00 | 0.00 | 0.00 | 0.00 | 0.00 | 0.00 | 1.37 | 0.00 |
| Number of keystone species | 0.00 | 0.00 | 0.00 | 3.00 | 3.00 | 3.00 | 3.00 | 3.00 | 3.00 | 3.00 |
| Robustness | 0.46 | 0.45 | 0.47 | 0.46 | 0.46 | 0.36 | 0.47 | 0.44 | 0.44 | 0.40 |
| Vulnerability | 0.34 | 0.41 | 0.34 | 0.27 | 0.34 | 0.60 | 0.26 | 0.56 | 0.57 | 0.75 |
| Total cohesion | 1.87 | 0.85 | 1.45 | 1.07 | 1.37 | 1.00 | 1.01 | 1.06 | 0.92 | 1.52 |
| Positive cohesion | 0.94 | 0.43 | 0.89 | 0.83 | 0.86 | 0.84 | 0.83 | 0.80 | 0.77 | 0.78 |
| Negative cohesion | -0.93 | -0.42 | -0.57 | -0.24 | -0.51 | -0.16 | -0.17 | -0.26 | -0.16 | -0.74 |
